# Supplementary material for: Assessing the size and growth of the US wetland and stream compensatory mitigation industry
Source: PLoS One. 2023 Sep 27;18(9):e0285139. doi: 10.1371/journal.pone.0285139 (PMC10529541; doi:10.1371/journal.pone.0285139)
Supplement: S1 File — (DOCX) [file pone.0285139.s001.docx]

**S1: Survey modifications and implementation**

Similar to BenDor et al.’s (2015) study, we began by asking respondents a screening question about their firms’ involvement in ecological restoration activities, generally. This was important to help us determine an accurate sample response rate, as the firms in our sampling design would not necessarily all be engaged in restoration activities (e.g., NMEMC conference attendees). As they did in 2014, negative responses to this screening question were used to adjust response rates (see Table 1) by removing non-respondent firms at the same rate as responses indicated non-involvement in restoration (i.e., assuming non-respondent firms were included in error at the same rate as responding firms indicated that their inclusion was in error).

Also keeping with the 2014 survey, respondents were asked to approximate their firm’s total revenue within a range (a dropdown list of 14 categories was provided representing ranges from “less than $100,000” to “over $500 Million“). To link these values to restoration, generally (and to provide a comparison to 2014 values), firms were then asked, “What percentage of your total revenue (sales) in your company or organization is derived from environmental restoration work?”

We modified and improved the 2014 survey in several key ways. Previously, respondents were asked about the “type” of environmental restoration work their firms engaged in, and were presented with a variety of common response categories they could select from (or write in their own). Unfortunately, while survey pre-tests at the time revealed universal understanding of these options, these response categories unintentionally blended references to specific ecosystems, legal drivers, ecological restoration activities, and whether or not these activities applied to popular mitigation frameworks (e.g., Clean Water Act, Section 404; Endangered Species Act).

For the present study, we have corrected this jumbling of legal drivers, ecosystems, activities, and mitigation by asking for revenue breakdowns around: 1) the role of mitigation in firms’ work, 2) the services they provided, 3) the ecosystem types they worked to restore, 4) the organizations that hired them, and 5) the federal, state, and local “drivers” of their restoration business (including [local, state, or federal] laws/statutes, regulations, policies, requirements, funding programs, incentives, or other reasons that cause you to restore ecosystems).

The survey was pre-tested with a variety of mitigation practitioners, including the ERBA Board of Directors, in May 2021. It was then distributed using the Qualtrics online survey platform between August 2021 and January 2022 (Qualtrics 2022; see Carpenter et al. 2019 regarding use prevalence of Qualtrics for research surveys). An introductory email was sent by the authors to all potential respondents introducing the project and its goals, providing questions ahead of time, and allowing firms to gather 2019 data. ERBA leadership also emailed its members encouraging them to respond, and sent along a follow-up reminder. As an incentive, ERBA offered all respondents $50, in the form of either a discount on annual membership dues or attendance to its annual professional conference.

Primary contacts for all firms were sent the survey, and non-respondents were sent reminders after several weeks (and at two-week intervals afterward, up to three reminders). If a firm’s primary contact did not respond, we then used the same procedure for secondary contacts. In a few cases (n=7 responses removed), we received belated responses from primary contacts in addition to secondary contacts (i.e., multiple, redundant responses from the same firm). In these cases, we kept only the most complete responses.
