# Supplementary material for: Assessing the size and growth of the US wetland and stream compensatory mitigation industry
Source: PLoS One. 2023 Sep 27;18(9):e0285139. doi: 10.1371/journal.pone.0285139 (PMC10529541; doi:10.1371/journal.pone.0285139)
Supplement: S2 File — (DOCX) [file pone.0285139.s002.docx]

**S2: Survey weighting**

A major part of estimating industry sizes and activities from a sample involves extrapolating the sample to the universe of firms in the industry. How do we estimate the economic impacts of the entire mitigation industry when not all firms within the industry will give us information about their activities and revenues? For example, what if only large, successful firms responded to our survey? Ultimately, we must evaluate whether the subset of respondents (sample) represent the entire industry.

While many surveys seek to correct response biases using various forms of “survey weighting,” whereby (sometimes) sophisticated multipliers are added to each response in an effort to reduce bias in the sample of responses (Biemer and Christ 2008). Unfortunately, in many cases, instead of reducing bias, complex survey weighting efforts act as opportunities for introducing bias (intentionally or unintentionally) into subsequent statistical analyses (Gelman 2007). Fortunately, we have no reason to believe that any systemic bias in the willingness of firms to respond was introduced during the survey creation or implementation process. Therefore, similar to the process used by BenDor et al. (2015), we assigned weights simply as the inverse of the probability of a response from a firm from each of the contact lists. In order to compare 2014 and 2019 response sets, we averaged this weight across these lists to create a single response weight for each survey year.
