# Supplementary material for: Assessing the size and growth of the US wetland and stream compensatory mitigation industry
Source: PLoS One. 2023 Sep 27;18(9):e0285139. doi: 10.1371/journal.pone.0285139 (PMC10529541; doi:10.1371/journal.pone.0285139)
Supplement: S3 File — (DOCX) [file pone.0285139.s003.docx]

**S3: Selecting 2014 mitigation firm subset and estimating margins of error**

As part of BenDor et al.'s (2015) effort to study the US ecological restoration sector, broadly, they defined two sampling frames for gathering survey data. The first, a *publicly-induced* restoration firm sample, aimed at capturing restoration performed in response to direct federal procurement or as mandated by public laws (e.g., US Clean Water Act) that require or induce mitigation. This unknown universe of actors was proxied using a portion of the 2012 database of government contractor firms listed on USASpending.gov (limited by contractors with federal agencies and industries known to be involved in restoration work; n=5,805 firms), which included many firms that worked directly on government contracts.

Their second sampling frame was aimed at the universe of restoration firms that worked for *private* *sector*-initiated restoration projects. This frame was created via a literature review and drew on listserves provided by the former NMBA (n=550 firms). Firms in both sampling frames were asked for contact information (referrals) of other firms that they believed were additionally involved in ecological restoration (thereby creating an additional “snowball sample”; (Parker, Scott, and Geddes 2019).

In total, BenDor et al. (2015) received 284 non-duplicate responses (out of 324 total), yielding adjusted sample response rates from the NMBA and public lists of 25.6 percent and 11.6 percent, respectively), which were used as inputs to the IMPLAN v.3.1 input-output model, yielding national estimates of restoration industry activities.

Importantly, for our purposes, BenDor et al. (2015) did not seek to define – or directly measure the activities of — the mitigation industry, specifically as driven by the US Clean Water Act. However, in light of the revised structure used for the present study, the 2014 survey does offer several pathways for retroactively establishing a subset of responses (and resulting survey weights) that facilitate comparison of the industry across 2014 and 2019 measurements. We explored a variety of scenarios, including subsets of only those firms (A) involved in mitigation banking (see BenDor et al. 2015; Supporting Information File S5, Q8 for the specific question), (B) involved in Clean Water Act Section 404 mitigation (in any manner; Q17), or C) that were part of the NMBA-provided contact list (and their referrals). We also looked at inclusive (“OR”) and intersecting (“AND”) sets of respondent firms for sets (A) and (B), above.

Our evaluations suggested that this latter, inclusive set (n=112 firms) – those firms indicating that they were involved in *either* mitigation banking or in Clean Water Act Section 404 mitigation – comprise a reasonably complete set of responses, representative of the mitigation industry at that time. Moreover, while the sampling frames we used in our 2021 survey were somewhat different, this 2014 firm subset can be replicated in our 2021 survey (see Supplementary Material 1 for the full 2021 survey instrument) by analyzing only firms involved in either Section 404 mitigation (Q15; n=51 firms) or in mitigation banking (Q23; n=56 firms). This selection reduces the 2019 sample of “mitigation-involved” firms to n=60 firms.

We can provide a broad assessment of the accuracy of our direct inputs by calculating the simple margin of error for each of the surveys as a whole, based on the sample sizes of survey respondents. Assuming that we drew a simple random sample for the universe of mitigation actors (both subsets of the broad group of respondents to each of our surveys), we can calculate the margin of error (with 95 percent confidence level) as Error = 1.96/[2∗sqrt(n)], which yields margins of error of 9.3 percent (2014) and 12.7 percent (2019), respectively.
