# Supplementary material for: Assessing the size and growth of the US wetland and stream compensatory mitigation industry
Source: PLoS One. 2023 Sep 27;18(9):e0285139. doi: 10.1371/journal.pone.0285139 (PMC10529541; doi:10.1371/journal.pone.0285139)
Supplement: S4 File — (DOCX) [file pone.0285139.s004.docx]

**S4: 2021 Survey Instrument**

You have been selected to participate in a study about the environmental restoration industry in the United States. This survey is a follow-up to our 2014 survey, which estimated the national economic impacts of environmental restoration. The goal of this study is to update our estimates of these impacts and understand how the restoration industry has changed since 2014.

All of your responses will be completely confidential and voluntary and only accessible for analysis by researchers at UNC-Chapel Hill. No other firms or individuals will be able to access your responses, ever. Your name will not be associated with any of your responses and you may quit the survey at any time. Your company or organization name will only be used internally by the research team to ensure that the same company is not represented more than once in the survey results. Your identifying information will not be shared with ERBA or any of the survey sponsors.

This survey contains about 25 questions, and will take 15 - 25 minutes of your time. You may choose to exit the survey at any time. As a thank you for your time and help, you will receive your choice of either a $50 discount for one registration for your company or organization at ERBA's 2021 Policy Conference (October 2021) or a $50 discount on your company or organization's 2022 ERBA membership dues. We will follow up regarding this discount after you complete this survey.

This project is led by Dr. Todd BenDor, Professor at the University of North Carolina, Chapel Hill. This survey has been approved by UNC's Institutional Review Board for the protection of human subjects (IRB# 13-1872) and the confidentiality of your responses is protected by strict federal law. If you have questions or comments about the survey, please email us at bendor@unc.edu.

Thanks very much.
Todd K. BenDor and T. William Lester

Department of City and Regional Planning

University of North Carolina Chapel Hill

Has your company or organization ever participated in any aspect of environmental restoration work, or worked with an environmental restoration company (i.e. supplier)?

*Yes*

*No*

*[Answering “No” will skip to the end of the survey]*

Please provide some basic information about your company or organization below.

1. What is the name of your company or organization?
2. In what state is your company or organization located? (If you have multiple locations, please select the location of the company headquarters.)

*[Respondents given list of states to select]*

1. In what year was your company or organization founded?
2. What is your company or organization's primary industry? Please indicate the North American Industry Classification (NAICS) code -- including Sector, Sub-sector, and Industry -- that best describes your line of work, if known. If you do not know the NAICS code, please select "Don't Know" in the Sector dropdown menu and provide a brief description of the line of work of your company organization in the following question.

*Sector*

*Sub-Sector*

*Industry*

[If “Don’t Know” answered] If you selected that you "Don't Know" your organization's NAICS code, please describe your company or organization's line of work.

A critical aspect of this survey is to estimate the total economic impact of environmental restoration work, nationwide. Therefore, it is essential that we ask about the total sales and employment levels at your company or organization. All of your responses are completely confidential.

We understand that 2020 may have been atypical for you because of COVID. Therefore, we ask that you base your responses on your sales and restoration activities from 2019.

1. What was the total revenue (sales) of your company or organization in 2019? Please include all revenue whether it is restoration-related or not. Please enter a number with no dollar sign. If you do not feel comfortable reporting a specific value, you can leave blank and report a range in the next question.

[If answer left blank] What was the total revenue (sales) of your company or organization in 2019? Please include all revenue whether it is restoration-related or not.

| *under $100,000 (1)*  *$100 - 200,000 (2)*  *$200- 500,000 (3)*  *$500,000 - $1 million (4)*  *$1 - $5 million (5)*  *$5 -$10 million (6)*  *$10- $20 million (7)* | *$20 - $40 million (8)*  *$40 - $60 million (9)*  *$60 - $80 million (10)*  *$100- $250 million (11)*  *$250 -$500 million (12)*  *more than $500 million (13)* |
| --- | --- |

1. With your best estimates, what percentage of your company or organization's total revenue (sales) in 2019 was derived from environmental restoration work? Please drag the slider bar to indicate the percentage.

|  | 0 | 10 | 20 | 30 | 40 | 50 | 60 | 70 | 80 | 90 | 100 |
| --- | --- | --- | --- | --- | --- | --- | --- | --- | --- | --- | --- |

| Percent of revenue from environmental restoration work | 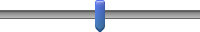 |
| --- | --- |

1. How many full-time employees are employed at your company/organization right now?
2. How many part-time employees are employed at your company/organization right now?
3. Does your company or organization's employment level change significantly throughout the year? (For example, if your company or organization hires seasonal employees to work on specific projects there would be a change in employment level over the course of one year.)

*Yes. It changes very significantly.*

*Yes. It changes somewhat significantly.*

*No. It does not change significantly.*

*[If “Yes” answered]* With your best estimates, what are the minimum and maximum number of people employed by your organization or company in 2019?

1. Please estimate the percentage of your total workforce within each level of educational attainment. All levels must total 100%

*Less than high school graduate*

*High school graduate or equivalent (GED)*

*Some college or associate's degree*

*Bachelor's degree*

*Graduate or professional degree*

1. Where does your organization or company engage in environmental restoration work? Select all that apply.

*[Respondents given list of states to select]*

Please tell us about the trends your company or organization is experiencing.

1. Over the last five years, has your company or organization's total revenue (sales):

*Increased*

*Decreased*

*Remained the same*

1. Over the last five years, has the share of your company or organization's total revenue (sales) that is related to environmental restoration:

*Increased*

*Decreased*

*Remained the same*

The following questions are going to ask about your company or organization’s restoration work. Again, when we use the word “you”, we are referring to your company or organization. We are going to ask questions about: 1) the role of mitigation in your work, 2) the services you provide, 3) the ecosystem types that you work to restore, 4) the organizations that hire you, and 5) the federal, state, and local “drivers” of your restoration business (which may be laws/statutes, regulations, policies, requirements, funding programs, incentives, or other reasons that cause you to restore ecosystems). It is essential to this survey that we collect information on the amount of your restoration business (sales) across each of these four categories. While you may not have this exact information, we hope that you will provide your best estimates for each of our questions.

1. Did your company or organization perform any restoration for mitigation purposes in 2019?

*Yes*

*No*

*[If “Yes” answered]*

1. What mechanisms did you use to provide mitigation in 2019?

| *Mitigation banking*  *Permittee responsible mitigation*  *In-lieu fee programs* | *Other, please describe*  *Other, please describe*  *Other, please describe* |
| --- | --- |

1. With your best estimates, please indicate the approximate percentage of your restoration-related sales that were due to each type of mitigation work in 2019. These percentages do not need to sum to 100% if not all of your restoration work was for mitigation purposes.

*[Carries forward choices from previous question]*

1. What are the types of environmental restoration-related services that your company or organization provided in 2019. Please include all of the services that your company or organization performed, whether in full or in part, related to environmental restoration projects. Do not include work that you never performed or always contracted out to other companies or organizations. Select all that apply.

*Legal Services*

*Project management (e.g., full service mitigation banking, restoration site implementation)*

*Financial Services*

*Credit sales or marketing*

*Real Estate/Site Acquisition*

*Planning and Permitting Services*

*Design and Engineering Services*

*Physical Restoration (for example, earth moving, planting, burning, removal of non-native species)*

*Monitoring, operations, or maintenance services*

*Supplies or equipment for landscaping or other physical restoration activities*

*Other supplies*

*Consulting services*

*Other services, please describe*

*Other services, please describe*

*Other services, please describe*

1. With your best estimates, please indicate the approximate percentage of your restoration-related sales due to each of the following activities in 2019 (You can indicate this for each activity. All activities must total 100%)

*[Carries forward choices from previous question]*

1. What ecosystem type(s) did your company or organization participate in restoring in 2019? Select all that apply.

*Wetland restoration and management (freshwater)*

*Wetland restoration and management (saltwater)*

*Freshwater stream or riparian/floodplain (non-coastal) restoration and management*

*Coastal, shoreline, estuarine, or marine restoration and management*

*Forest restoration*

*Grassland restoration*

*Enhanced stewardship (e.g. sustainable timbering, ranching, farming)*

*Developed or formerly developed sites (e.g. environmental clean ups and contamination management)*

*Other, please describe*

*Other, please describe*

*Other, please describe*

1. With your best estimates, please indicate the approximate percentage of your restoration-related sales due to your restoration work in each of these ecosystem types in 2019 (You can indicate this for each activity. All activities must total 100% )

*[Carries forward choices from previous question]*

1. What types of organizations typically hire your company or organization to do restoration work? Select all that apply.

*Private company*

*Federal government agency*

*State or local government agency*

*Non-governmental organization (NGO)*

*Other, please describe*

*Other, please describe*

*Other, please describe*

1. With your best estimates, please indicate the approximate percentage of your restoration-related sales to each of the entities that hired your company or organization to do restoration work in 2019 (You can indicate this for each activity. All activities must total 100% )

*[Carries forward choices from previous question]*

1. What are the federal legal drivers of your organization's restoration-related work? Select all that apply

| - *Clean Water Act Section 404 (including wetland and stream mitigation)* - *Endangered Species Act* - *Clean Water Act Section 402/Water Quality or Nutrient Trading* - *Greenhouse gas (GHG) or carbon offset markets* - *Federal Highway Administration (FHWS) Mitigation or restoration related to Executive Order 11990 (Protection of Wetlands)* - *Natural Resource Damage Assessment (NRDA)* - *Comprehensive Environmental Response, Compensation, and Liability Act (CERCLA* - *Resource Conservation and Recovery Act (RCRA)* - *Federal Emergency Management Agency (FEMA) programs* - *Abandoned Mine Lands Program* - *Natural Resource Conservation Service Program (for example, the Environmental Quality Incentives Program)* | - *Farm Service Agency program (for example, the Conservation Reserve Program)* - *U.S. Fish and Wildlife Service (USFWS) program (for example, North American Wetlands Conservation Act programs)* - *National Oceanic and Atmospheric Administration (NOAA) program (for example, Community-based Restoration grants)* - *U.S. Forest Service (USFS) program (for example, Stewardship Contracting)* - *Bureau of Reclamation (for example, the WATERSmart program)* - *Oil Pollution Act* - *National Fish and Wildlife Foundation federally-funded programs* - *Other, please describe* - *Other, please describe* - *Other, please describe* |
| --- | --- |

1. Were there any state or local legal drivers of your restoration-related work in 2019? For example, these may include pay-for performance-contracts, state bonds, tax credits, municipal solid waste projects, or other drivers.

*Yes*

*No*

*[If “Yes” answered]* Please list each of these local and state restoration drivers. For each driver, please be as specific as possible about: 1) the formal names of these drivers (e.g., the name of the law, regulation, or funding program) 2) the geographic locations where these drivers were in effect.

*[Respondents can list legal drivers and their locations]*

| *Legal Driver* | *Location* |
| --- | --- |
|  |  |

1. With your best estimates, please indicate the approximate percentage of your restoration-related sales that were due to each of the drivers of your restoration work in 2019. (You can indicate this for each activity. All activities must total 100% )

*[Federal and local/state restoration drivers are carried forward from previous questions]*

1. The following question only pertains to your company or organization's restoration activities. 28) Currently, are there any major barriers or impediments to gaining financial investments for your company or organization?

*Yes*

*No*

*[If “Yes” answered]* Please tell us with as much detail as possible about each barrier and its source, and offer specific examples, if possible.

1. If you know of any other person/company who would be interested in taking this survey to help us get additional information, please enter their contact information below. Thank you very much!

*[Respondent can input Name, Email Address, and Company/Organization for four (4) individuals]*
